# Long Short-Term Memory Network for Development and Simulation of Warfarin Dosing Model Based on Time Series Anticoagulant Data

**DOI:** 10.3389/fcvm.2022.881111

**Published:** 2022-05-11

**Authors:** Yun Kuang, Yaxin Liu, Qi Pei, Xiaoyi Ning, Yi Zou, Liming Liu, Long Song, Chengxian Guo, Yuanyuan Sun, Kunhong Deng, Chan Zou, Dongsheng Cao, Yimin Cui, Chengkun Wu, Guoping Yang

**Affiliations:** ^1^Center of Clinical Pharmacology, The Third Xiangya Hospital, Central South University, Changsha, China; ^2^XiangYa School of Pharmaceutical Sciences, Central South University, Changsha, China; ^3^Department of Pharmacy, The Third Xiangya Hospital, Central South University, Changsha, China; ^4^School of Mathematics and Statisics, Central South University, Changsha, China; ^5^Department of Cardiovascular Surgery, The Second Xiangya Hospital, Central South University, Changsha, China; ^6^Hunan Key Laboratory of Diagnostic and Therapeutic Drug Research for Chronic Diseases, Central South University, Changsha, China; ^7^Institute of Clinical Pharmacology, Peking University First Hospital, Beijing, China; ^8^Department of Pharmacy, Peking University First Hospital, Beijing, China; ^9^State Key Laboratory of High Performance Computing, Institute for Quantum Information, College of Computer Science and Technology, National University of Defense Technology, Changsha, China; ^10^National-Local Joint Engineering Laboratory of Drug Clinical Evaluation Technology, Changsha, China

**Keywords:** long short-term memory network, modeling, warfarin, time series, application

## Abstract

**Background:**

Warfarin is an effective treatment for thromboembolic disease but has a narrow therapeutic index, and dosage can differ tremendously among individuals. The study aimed to develop an individualized international normalized ratio (INR) model based on time series anticoagulant data and simulate individualized warfarin dosing.

**Methods:**

We used a long short-term memory (LSTM) network to develop an individualized INR model based on data from 4,578 follow-up visits, including clinical and genetic factors from 624 patients whom we enrolled in our previous randomized controlled trial. The data of 158 patients who underwent valvular surgery and were included in a prospective registry study were used for external validation in the real world.

**Results:**

The prediction accuracy of LSTM_INR was 70.0%, which was much higher than that of MAPB_INR (maximum posterior Bayesian, 53.9%). Temporal variables were significant for LSTM_INR performance (51.7 vs. 70.0%, *P* < 0.05). Genetic factors played an important role in predicting INR at the onset of therapy, while after 15 days of treatment, we found that it might unnecessary to detect genotypes for warfarin dosing. Using LSTM_INR, we successfully simulated individualized warfarin dosing and developed an application (AI-WAR) for individualized warfarin therapy.

**Conclusion:**

The results indicate that temporal variables are necessary to be considered in warfarin therapy, except for clinical factors and genetic factors. LSTM network may have great potential for long-term drug individualized therapy.

**Trial Registration:**

NCT02211326; www.chictr.org.cn:ChiCTR2100052089.

## Introduction

Warfarin is an effective anticoagulant and is the most commonly used anticoagulant to prevent and treat thromboembolic disease worldwide (1). The effect of warfarin is measured with a blood coagulation index known as the international normalized ratio (INR) (2). Patients with different diseases taking warfarin have different target INR ranges (e.g., 1.8–2.3, 2.0–2.5, or 2.0–3.0). However, a particular challenge with the use of warfarin is its narrow therapeutic index, associated with large individual variations in daily dose requirements and often leading to either bleeding or thrombosis (3). This has led warfarin to becoming one of the top 10 drugs associated with drug-related hospitalization (4). Thus, warfarin doses required to achieve the target INR ranges are determined via several days of testing, which leads to frequent return visits and blood draws.

A potential strategy for improve warfarin efficacy and safety is to account for individual genetic variations of *CYP2C9* and *VKORC1*, along with clinical variations (5). Various warfarin dosing algorithms have been reported to predict warfarin dosing; International Warfarin Pharmacogenetics (IWPC) (6), Gage et al. (7), Lenzini et al. (8), and Lee et al. (9–12) have developed some predictive formulas for personalized warfarin dosing to improve anticoagulation control. However, these models only have about 50% prediction accuracy. Almost all existing algorithms only cross-sectionally consider the effects of various fixed variables on warfarin dosing, ignoring time series variables, meaning that these models fail to reflect real-world anticoagulation therapy.

Our multicenter, randomized, single-blind, parallel-controlled trial called XY3-WAR (Warfarin Trial of the Third Xiangya Hospital, Central South University) (13) demonstrated the utility of genotype-guided dosing of warfarin to optimize individual warfarin dosing in a Chinese population. The trial included 660 patients with deep vein thrombosis and atrial fibrillation who were treated with warfarin for 3 months. Those data were high-quality time series anticoagulant data. Long short-term memory (LSTM) has a powerful capacity for processing long temporal data and solving the problem of long-term dependencies (14). With the development of big data in the medical field, LSTM networks have been increasingly used for various medical tasks. Maragatham used patients' electronic medical records to propose a heart failure prediction model based on LSTM networks, which resulted shown improved accuracy (15). Syed Hasib used LSTM networks for forecasting dynamic blood glucose levels of patients with type 2 diabetes mellitus (16). These successful attempt inspired us that LSTM network might be a potential strategy for warfarin individualized therapy.

This study first aimed to adopt LSTM networks to develop an individualized INR model based on the time series anticoagulant data from the XY3-WAR trial, then use external data from a prospective study to evaluate this model in the real world. The study's secondary aim was to simulate warfarin dosing individualization using the LSTM_INR model we developed.

## Materials and Methods

### Study Design and Population

The modeling data set was the time series anticoagulation data of the XY3-WAR study (13). We excluded patients who did not take warfarin or were lost follow-up on day 4/5. Finally, a total of 624 patients with atrial fibrillation or deep vein thrombosis were enrolled in the modeling data set. The external validation data set was from a prospective registry study, and a total of 158 volunteers were enrolled (from the Second Xiangya Hospital of Central South University, Changsha, China). The detailed inclusion and exclusion criteria are available in the Supplementary Methods.

### Ethical Considerations

The XY3-WAR study (NCT02211326) was approved by the Institutional Review Board of the Third Xiangya Hospital of Central South University, and the institutional review boards of each participating hospital. The prospective registry study (www.chictr.org.cn:ChiCTR2100052089) was approved by the Institutional Review Board of the Second Xiangya Hospital of Central South University. All subjects provided written informed consent.

### Genetic Analysis

Genotyping for the CYP2C9^*^2, CYP2C9^*^3, and *VKORC1-1639G*>*A* alleles was performed using the amplification refractory mutation system. More information on genotyping is available in the Supplementary Methods.

### Model Development

**Figure 1:**
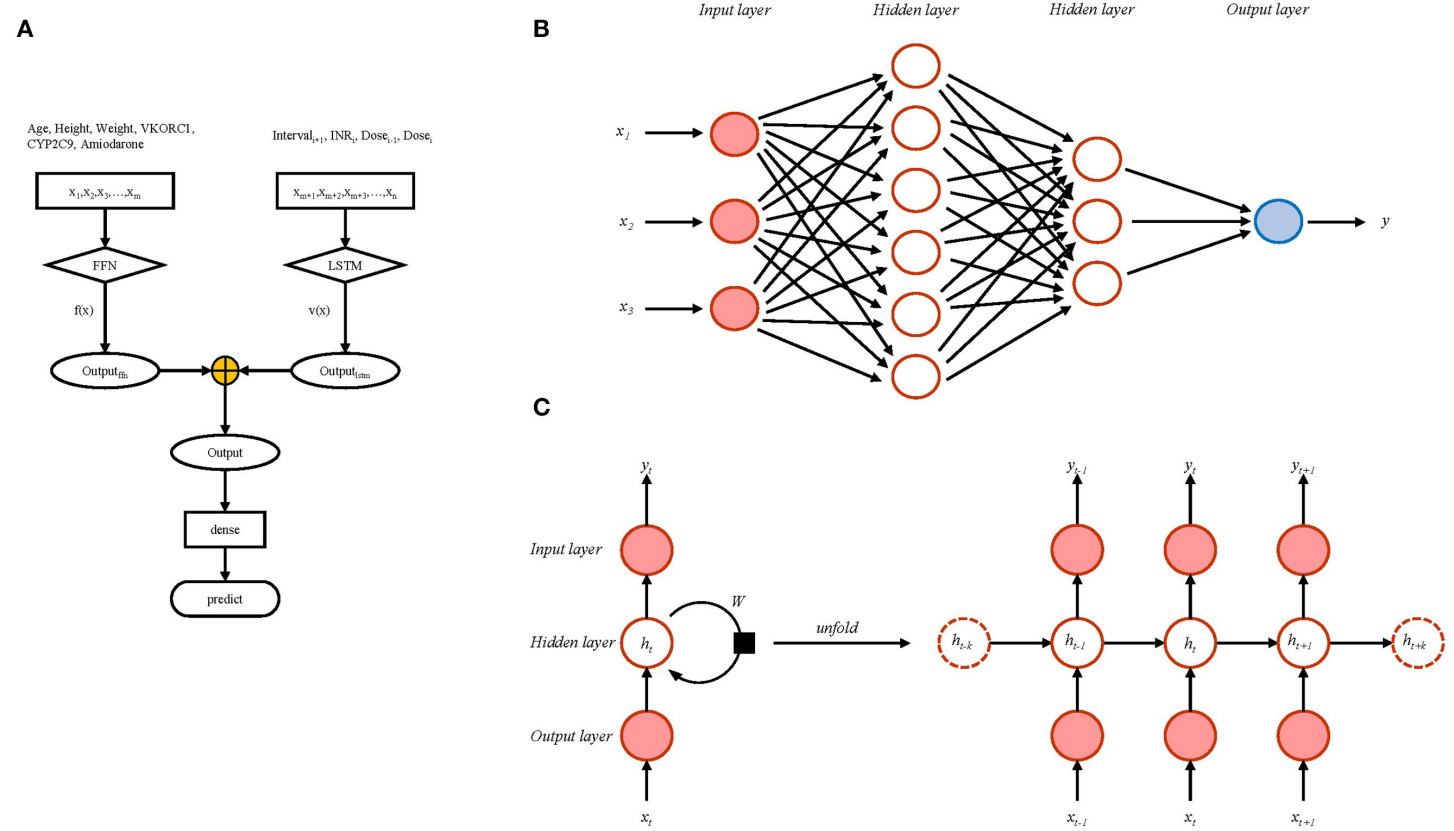
The modeling flow chart **(A)** including schematic representation of feedforward neural network **(B)** and long short-term memory network **(C)**.

According to the modeling data set of the XY3-WAR study, we included age, height, weight, *VKORC1, CYP2C9*, and amiodarone status data reported in the research; these were defined, fixed variables. The defined time series variables were adjusted dose at last follow-up (Dose_i−1_), INR at this follow-up (INR_i_), interval from this follow-up to the subsequent follow-up (Interval_i+1_), and adjusted dose at this follow-up (Dose_i_). Fixed variables were processed using a feedforward neural network (FNN), and time series variables were processed using LSTM. The output was INR at next follow-up. The detailed modeling flow chart is shown in Figure 1A. FNNs are among the most basic forms of artificial neural networks with the simplest structure and are used successfully in many applications. The data from the input layer were processed by hidden layers, then processed data were transferred to the output layer to finally generate results. The whole process proceeded in a single direction (Figure 1B). LSTM networks are recurrent neural networks that capture both long-term and short-term dependencies within sequential data. Differing from the single-direction transfer of FNNs, many of the units of LSTM networks are linked together in time series (Figure 1C).

Formula (1) was used for data standardization; 10% of the modeling data set was randomly set as the test set, and the remaining modeling data set was divided into a training set (90%) and a validation set (10%). We set the mean square error (MSE) (2) as the loss function of neural networks. The batch size was set to 64, the learning rate was 0.001, and the epoch was 100. To prevent the model from overfitting, we used early stopping for regularization. The stopping criteria were based on the loss function, and training stopped when the loss function did not decrease after 10 epochs. We used 10-fold cross-validation to train the model. Mean absolute error (MAE) (3), root mean square error (RMSE) (4), and prediction accuracy were used to evaluate the stability of the model. We exported the model at the last time as the final model. A flow chart of the analysis used for model development and model validation is shown in Supplementary Figure S1.


(1) $$\begin{array}{r} x_{i}=\frac{x_{i}-\overset{\bar{}}{x}=\frac{1}{n}\sum_{1}^{n}x_{i}}{s=\sqrt{\frac{1}{n-1}\sum_{1}^{n}{\;\left(x_{i}-\overset{\bar{}}{x}\right)}^{2}}} \end{array}$$

(2) $$\begin{array}{r} loss=\frac{\sum_{i=1}^{n}{\left({ŷ}_{i}-y_{i}\right)}^{2}}{n} \end{array}$$

(3) $$\begin{array}{r} MAE=\frac{1}{n}\overset{n}{\underset{n=1}{\sum }}|{ŷ}_{i}-y_{i}| \end{array}$$

(4) $$\begin{array}{r} RMSE=\sqrt{\frac{1}{n}\overset{n}{\underset{n=1}{\sum }}{\left({ŷ}_{i}-y_{i}\right)}^{2}} \end{array}$$

### Model External Validation

To evaluate the LSTM_INR model, we conducted a prospective registry study at the Second Xiangya Hospital of Central South University from 2020-12-29 to 2021-08-31. The follow-up visit data of patients we enrolled were used as the external validation data set. The evaluation variable was prediction accuracy (defined as within 70–130% of the true value). SPSS Statistics for Windows, version 25.0 (IBM Corp., Armonk, NY, USA) was used for statistical analysis. We used chi-square analysis to compare the prediction accuracy of various models; *P-*values <0.05 were considered to be statistically significant. This study compared the prediction accuracy of the LSTM models we developed with maximum posterior Bayesian (MAPB) models developed by Hamberg et al. (11). Furthermore, we investigated the effects of temporal variables on INR prediction and genetic factors at different follow-up periods. Subgroup analysis was used to compare the prediction accuracy of the LSTM model for patients with different sensitivities to warfarin. Finally, we simulated individualized warfarin dosing based on LSTM_INR and developed an application (AI-WAR) for warfarin individualized therapy.

## Results

### Participants

A total of 624 participants were enrolled in the modeling data set. The mean weight and height were 62.0 ± 12.2 kg and 161.8 ± 8.1 cm, respectively. Among the enrolled participants, 86.7% had atrial fibrillation, and the remainder (13.3%) had deep vein thrombosis. There were 316 (50.6%) male participants. The frequency of *VKORC1 AA, VKORC1 GA*, and *VKORC1 GG* were 80.1, 18.1, and 1.8%, respectively. The frequency of CYP2C9^*^1^*^1 was 92.8%, which was much higher than those of CYP2C9^*^1^*^3 (6.9%) and CYP2C9^*^3^*^3 (0.3%).

For the external validation data set, 158 participants were enrolled. The mean age was 53.1 ± 10.3 years. The mean weight and height were 60.2 ± 9.1 kg and 161.4 ± 7.5 cm, respectively. All participants had undergone cardiac valve surgery: 50.6% of patients had undergone mechanical valve replacement, 43.7% patients had undergone biological valve replacement, and 5.7% had undergone valvuloplasty. In this data set, 42.4% of participants were male. The frequencies of *VKORC1 AA* and *VKORC1 GA* were 88.0 and 12.0%, respectively, and those of CYP2C9^*^1^*^1 and CYP2C9^*^1^*^3 were 89.2 and 10.8%, respectively. Details of demographic and genetic information are shown in Table 1.

**Table 1:** Demographic and clinical information of modeling dataset and external validation dataset.

**Characteristics**	**Modeling set *n* = 624**	**External validation set *n* = 158**	***P***
	**Mean ±SD/*n* (%)**	**Mean ±SD/*n* (%)**	
**Sex**			0.03^*^
Male	316 (50.6%)	67 (42.4%)	
Female	308 (49.4%)	91 (57.6%)	
Age	67.5 ± 10.2	53.1 ± 10.3	<0.001^†^
Height (cm)	161.8 ± 8.1	161.4 ± 7.5	0.65^†^
Weight (kg)	62.0 ± 12.2	60.2 ± 9.1	0.045^†^
**Indication**			
Atrial fibrillation	541 (86.7%)	0 (0.0%)	
Deep vein thrombosis	83 (13.3%)	0 (0.0%)	
Mechanical valve displacement	0 (0.0%)	80 (50.6%)	
Biological valve displacement	0 (0.0%)	69 (43.7%)	
Valvuloplasty	0 (0.0%)	9 (5.7%)	
Follow-up time (day)	51.8 ± 23.5	72.1 ± 62.1	0.04^†^
%TTR^§^	52.5 ± 27.3	27.03 ± 23.36	<0.001^†^
Amiodarone use	9 (1.4%)	12 (7.6%)	0.001^*^
***VKORC1***			0.04
A/A	500 (80.1%)	139 (88.0%)	
A/G	113 (18.1%)	19 (12.0%)	
G/G	11 (1.8%)	0 (0.0%)	
***CYP2C9***			0.21
^*^1/^*^1	579 (92.8%)	141 (89.2%)	
^*^1/^*^3	43 (6.9%)	17 (10.8%)	
^*^3/^*^3	2 (0.3%)	0 (0.0%)	
**Sensitivity**			0.02
Highly sensitive responders	34 (5.5%)	15 (9.5%)	
Sensitive responders	477 (76.4%)	126 (79.7%)	
Normal responders	113 (18.1%)	17 (10.8%)	

* *χ^2^ test*.

† *Kruskal-Wallis test*.

*Continuity correction χ^2^ test*.

§ *The percentage of time in the therapeutic range*.

### Model Development

Trend graphs of loss function for 10-fold cross-validation were shown in Supplementary Figure S2. MSE, MAE, RMSE, and mean prediction accuracy are shown in Supplementary Table S1. The loss function trends for each model were the same—convergence to ~0.48 triggered early stopping. MAE was 0.5 ± 0.007 and MSE was 0.7 ± 0.01, which was slightly higher than convergence of loss function. RMSE was 0.8 ± 0.008, and prediction accuracy reached up to 70.6 ± 1.1%. There were slight variations among the evaluation parameters, indicating that this modeling process was stable. The final LSTM_INR model was the last modeling one; MAE was 0.5, MSE was 0.7, RMSE was 0.8, and prediction accuracy was 71.6%.

### Model External Validation

#### Comparison of LSTM_INR With MAPB_INR

**Figure 2:**
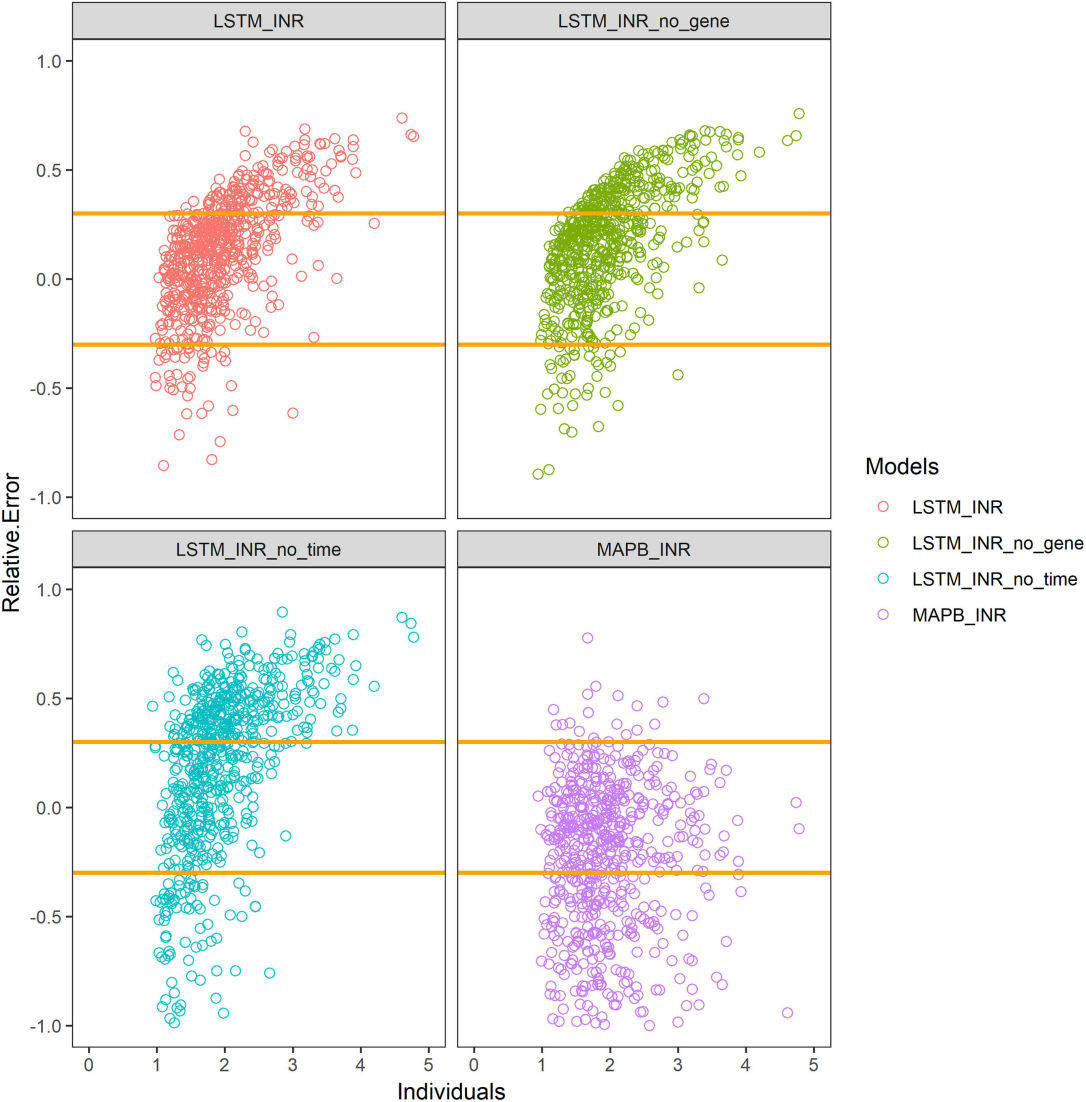
Prediction accuracy of different models. LSTM_INR_no_gene means LSTM_INR model without genotype data; LSTM_INR_no_time means LSTM_INR model without temporal data; MAPB_INR means INR model based on maximum posterior Bayesian. Two yellow lines show the range of 70–130% of true values. Plots above the line means overestimated, plots under the line means underestimated.

The results are shown in Table 2 and Figure 2. The prediction accuracy of the LSTM_INR model was up to 70.0%, while that of the MAPB_INR model was only 53.9% (*P* < 0.05), indicating that the LSTM_INR model performed significantly better than the MAPB_INR model.

**Table 2:** Prediction accuracy of different models.

**Model**	**Prediction accuracy (%)**	***P***
LSTM_INR	70.0% (462/660)	
MAPB_INR	53.9% (356/660)	<0.001^*^
LSTM_INR_no_time	51.7% (341/660)	<0.001^†^
LSTM_INR_no_gene	61.5% (406/660)	<0.001

* *LSTM_INR vs. MAPB_INR*.

† *LSTM_INR vs. LSTM_INR_no_time*.

*LSTM_INR vs. LSTM_INR_no_gene*.

#### The Effect of Temporal Variables

We, respectively, inputted Dataset_N (normal external validation data set) and Dataset_T (data set without temporal sequence) into LSTM_INR, and the results are shown in Table 2 and Figure 2. After removing temporal sequence, the prediction accuracy of LSTM_INR dropped to 51.7% (*P*<*0.05*).

#### The Effect of Genetic Factors at Different Follow-Up Periods

**Figure 3:**
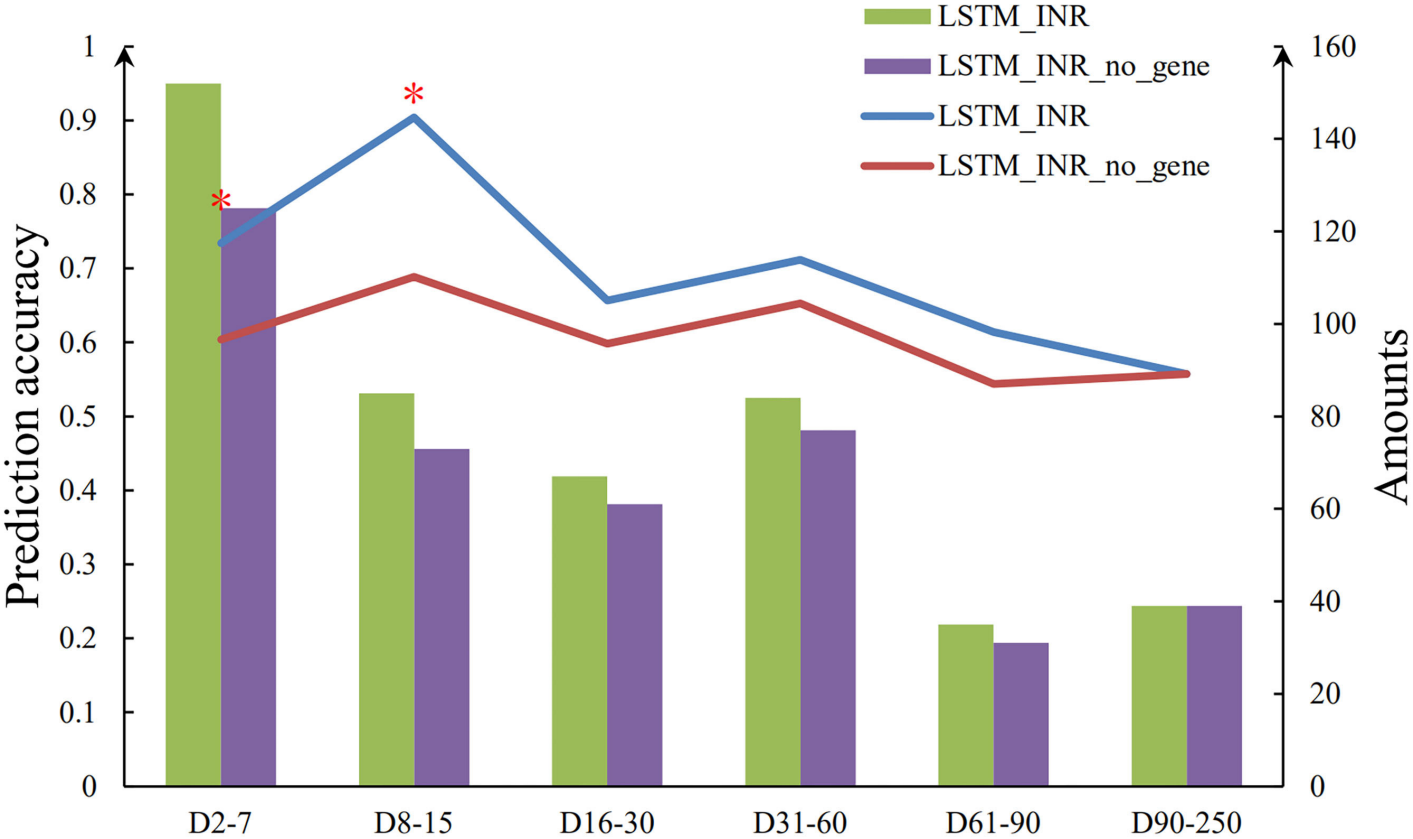
Sequence diagram of effect of genetic factors on LSTM_INR. *There are significant differences between two groups (*P* < 0.05).

As shown in Table 2 and Figure 2, the prediction accuracy of LSTM_INR dropped by 8.5% without regard to genetic factors (*P* < 0.05). At the onset of anticoagulant therapy, the prediction accuracy of LSTM_INR was significantly higher than that of LSTM_INR_no_gene (without genetic factors). After 15 days of treatment, the difference in prediction accuracy between LSTM_INR and LSTM_INR_no_gene was not statistically significant. Details are shown in Supplementary Table S2 and Figure 3. Regardless of genetic factors, prediction accuracy increased with time during the initial 15-day therapy period, while after day 15, prediction accuracy dropped gradually.

#### Prediction Accuracy of Subgroups With Different Genotypes

Additional subgroup analyses for different *CYP2C9* and *VKORC1* genotypes were performed. Three groups were defined based on the Food and Drug Administration genotype-based dosing recommendations as follows:

- Highly Sensitive responder: CYP2C9^*^1/^*^3 and VKORC1 AA, CYP2C9^*^3/^*^3 and *VKORC1 AA* or *GG* or *GA*
- Sensitive responder, CYP2C9^*^1/^*^1 and VKORC1AA, CYP2C9^*^1/^*^3 and *VKORC1 GG* or *GA*
- Normal responder: CYP2C9^*^1/^*^1 and *VKORC1 GG* or *GA*

We evaluated the prediction accuracy of LSTM_INR among these three subgroups. The results are shown in Supplementary Table S3, and the normal responder group has the highest accuracy of 75.7%, compared with the sensitive responder group (70.0%) and highly sensitive group (61.7%).

#### Simulation of Individualized Warfarin Dosing

For improved application of clinical anticoagulant therapy, we intended to predict individualized warfarin dosing based on LSTM_INR. Target INR ranges varied by treatment indication. In our external validation data set, target INR ranges were as follows: 1.8–2.3 for patients with mechanical valve replacements, 2.0–2.5 for patients with biological valve replacements, and 1.5–2.5 for patients who had undergone valvuloplasty.

Since the most common specifications of warfarin in the market were 2.5 mg/tablet and 3 mg/tablet, we inputted 16 possible prescribed doses (0.625, 0.75, 1.25, 1.5, 1.875, 2.25, 2.5, 3, 3.125, 3.75, 4.375, 4.5, 5, 5.25, 5.625, and 6 mg) into LSTM_INR, set target INR ranges for individuals, and then model-predicted INR were outputted. The final optimal individualized warfarin doses we recommended corresponded to the predicted INR within the target INR ranges (Supplementary Figure S3).

We developed an application called AI-WAR (Supplementary Figure S4), in which we embedded the LSTM_INR model for better warfarin therapy. AI-WAR offered dose decisions based on LSTM_INR, and clinicians could choose to accept the dose recommendation or input their prescriptions manually. Patients then could receive the final prescribed daily dose via the AI-WAR. More details about AI-WAR were shown in Supplementary Material.

## Discussion

Based on the powerful capacity of LSTM to process time series data, we successfully used a time series anticoagulant data set to develop our LSTM_INR model. The prediction accuracy with the test set was 71.6%. Lee et al. (9) proposed the use of LSTM networks to develop an INR prediction model to predict INR_day5_ for Korean patients. This study confirmed that LSTM can be applied to predict INR. However, the in-hospital INR_day5_ has minimal significance in long-term anticoagulant therapy. Besides, the study by Lee et al. (9) was limited in that it lacked prospective evaluation. LSTM_INR we developed was to predict INR of the next visit, which is appropriate for short - and long-term anticoagulant therapy.

For further evaluation of LSTM_INR in the real world, we conducted a prospective study for external validation. With the external validation data, we compared the prediction accuracy of LSTM_INR with MAPB_INR. MAPB_INR was developed by Bayesian forecasting and was proved effective and accurate in some studies. Based it, Hamberg developed a dose decision platform (WarfarinDoseCalculator) (17). This platform was widely used for individualization of warfarin (18). However, LSTM_INR performed better than MAPB_INR (70.0 vs. 53.9%, *P* < 0.05). Then, we evaluated the influence of temporal variables on the model, finding that the inclusion of temporal variables significantly increased the accuracy of LSTM_INR (70.0 vs. 51.7%, *P* < 0.05). This indicated that temporal variables are particularly useful in individualized INR prediction.

Given that it is not easy to obtain genotypes in primary hospitals in China, we evaluated the effect of genetic variables on LSTM_INR. LSTM_INR had better prediction accuracy than LSTM_INR_no_gene (70.0 vs. 61.5%, *P* < 0.05). Although the difference was statistically significant, the prediction accuracy of LSTM_INR_no_gene was still as high as 61.5%, which indicated that it is appropriate for use in primary hospitals. We also analyzed the effects of genetic variables in different follow-up subgroups, and we found that genetic factors were important for INR prediction at the initial therapy period (days 1–15), while after 15 days of therapy, the effects of genetic factors were minimal. This result aligned with previous observations (19, 20). The impact of gene polymorphisms on the initial phase of warfarin therapy was higher than that on the maintenance phase. Our results suggested that it was necessary to detect genotypes for patients at the initiation of therapy, while for patients who had more than 15 days of therapy, clinical factors and temporal variables were enough to predict individualized INRs. These results suggest that LSTM-INR would be useful for patients initiating anticoagulation therapy; for patients who have been on warfarin for 15 days, LSTM_INR_no_gene can be used. Warfarin has a slow anticoagulant effect. After 3–5 days of taking warfarin at the first time, INR would have significant fluctuations (21). In that case, LSTM_INR outperformed at the period of Day_7−15_ than Day_2−7_. Besides, all participants enrolled in XY3-WAR were asked to have regular INR testing during 12 weeks. The follow-up rate as follows: baseline test, 1, 4/5, 8 ± 1 (476/624), 15 ± 1 (421/624), 22 ± 1 (375/624), 28 ± 2 (460/624), 57 ± 3 (399/624), and 87 ± 3 days (38/624). During the D_0−60_, most of patients (over 60%) test INR on time, while after 60 days, a great number of patients dropped out. In the external validation datasets, the rate of regular follow-up was 90.8% from days 1–15, but it was 45.38% after day 15. As results shown, LSTM_INR performed excellent during the early period (D_1−15_) but the accuracy decreased at the time of D_15_ and D_60_. There were two main reasons explained it. One was that the follow-up time of external validation dataset were not as regular as modeling dataset. Another was that the sample size of long-term follow-up (>60 days) in the modeling dataset decreased. So that LSTM_INR could not performed great after Day_60._

Many models have been developed for predicting INR or individualized dosing for warfarin therapy. However, the relevant studies have only focused on the initial (10) and the maintenance (22) phases. In the real world, patients who take warfarin require long-term or even life-long anticoagulant therapy, so these preexisting models were not applicable for adjusting warfarin doses throughout long-term follow-up. Besides, most models only considered cross-sectional data and ignored the effects of temporal variables. The LSTM_INR model we developed aimed to predict the INR of the next visit, which was more appropriate for real-world anticoagulation scenarios. Beyond that, our modeling data set enrolled patients with atrial fibrillation and deep vein thrombosis, while the external validation data set enrolled patients after valvular surgery. The prediction accuracy of this model was still about 70%, indicating that this model was applicable to various indications.

Unlike previous studies, our study further performed subgroup analyses by genotype. The prediction accuracy associated with the normal responder group was higher than those associated with sensitive responder and highly sensitive responder groups. This result was in accordance with our previous study (XY3-WAR). In China, most people are sensitive responders; thus, clinicians prescribe as if the entire population comprises sensitive responders, which can lead to thrombosis for normal responders and bleeding for highly sensitive responder. Thus, our LSTM-INR was beneficial for the normal responder group.

The accuracy we defined in this study was acceptable for clinical use (23). However, for better supporting clinical decision-making, we further evaluated the prediction accuracy of LSTM_INR within ±20%. There was a no doubt that accuracy decreased within the range of ±20%. Finally, LSTM_INR only got the accuracy of 53.93%. But LSTM_INR still has significant performance than MAPB method. Further, we evaluated the accuracy in different subgroups. We found LSTM_INR has better performance in low INR range ( ≤ 2) with the accuracy of 57.87% (239/413). This result indicated LSTM_INR would be more suitable for real-world treatment of low-intensity anticoagulation in China (24–26). Many experts recommended the ideal INR range was 1.8–2.0 in Chinese patients underwent heart valve replacement. Besides, we found the accuracy of model during D_7−15_ was the highest (59.43%), which was corresponding to our study with ±30% range. In that case, we believed LSTM_INR has clinical significance in supporting decision-making for Chinese patients in low-intensity anticoagulation management, especially at the initial 15 days.

To assist clinicians with individualizing warfarin therapy, we simulated individualized warfarin dosing based on LSTM_INR. The trend of using mobile applications or electronic systems for anticoagulation is likely more suitable for long-term individualized therapy and anticoagulant self-management (27, 28). Thus, we developed an application (AI-WAR) in which we embedded these were embed in. Unlike other mobile anticoagulant systems, the critical function of AI-WAR was dose decision support. This function could help clinicians improve individualized therapy. In the future, we will conduct a prospective study to evaluate the clinical efficacy of AI-WAR in the real world.

Our study had some limitations. First, this study only recruited participants from Hunan province. However, given that the racial distribution in Hunan province is similar to that of China overall, the participants who enrolled in our study can be considered representative of the Chinese population (29). Second, the external validation data set was from a prospective registry study, and the INR of most subjects were not monitored regularly, while the modeling data set was from a randomized controlled trial for which nearly all subjects needed to be followed up at specified times. Thus, the prediction accuracy of models before day 30 were higher than that after day 30. It is worth mentioning that in this case, the prediction accuracy of the models that we developed were still about 60%.

## Conclusion

This study innovatively incorporated temporal variables into LSTM networks to develop an individualized INR prediction model and successfully simulated individualized warfarin dosing, which can be applied for adjusting the warfarin doses of patients throughout long-term follow-up.

## Data Availability Statement

The original contributions presented in the study are included in the article/Supplementary Material, further inquiries can be directed to the corresponding author/s.

## Ethics Statement

The studies involving human participants were reviewed and approved by the Institutional Review Board of the Third Xiangya Hospital of Central South University. The patients/participants provided their written informed consent to participate in this study.

## Author Contributions

YK, YL, and QP wrote the first version of the manuscript. YK, QP, CG, and GY designed and supervised this study. YZ and CW constructed models. LS, LL, YL, and XN performed the clinical trial. YL, XN, YS, and KD collected and analyzed data. CZ, DC, and YC revised the manuscript for important content. All authors agreed the final draft of the manuscript.

## Funding

This research was supported by the Key Research and Development Project of Hunan Province (2020SK2010) and National Major New Drug Creation Project of China (No. 2020ZX09201-010).

## Conflict of Interest

The authors declare that the research was conducted in the absence of any commercial or financial relationships that could be construed as a potential conflict of interest.

## Publisher's Note

All claims expressed in this article are solely those of the authors and do not necessarily represent those of their affiliated organizations, or those of the publisher, the editors and the reviewers. Any product that may be evaluated in this article, or claim that may be made by its manufacturer, is not guaranteed or endorsed by the publisher.
