# Supplementary material for: Long Short-Term Memory Network for Development and Simulation of Warfarin Dosing Model Based on Time Series Anticoagulant Data
Source: Front Cardiovasc Med. 2022 May 11;9:881111. doi: 10.3389/fcvm.2022.881111 (PMC9130657; doi:10.3389/fcvm.2022.881111)
Supplement: Supplementary file 1 [file Data_Sheet_1.docx]

Supplementary Material

# Supplementary Methods

## Registry study([www.chictr.org.cn:ChiCTR2100052089](http://www.chictr.org.cn:ChiCTR2100052089))

### Inclusion criteria

1. Patients who will take or are taking warfarin;
2. Patients who can be followed up at least once;
3. Patients who are able to provide written informed consent and communicate well with the investigator and complete the study in accordance with the study regulations.

### Exclusion criteria

1. Patients whose medical records cannot be provided or are incomplete.

## Genetic analysis

The techniques of amplification refractory mutation system (ARMS) and pyrophosphoric acid sequencing are employed for detecting the gene polymorphisms of *CYP2C9*（rs1799853）& (rs1057910) and *VKORC1* (rs9923231).

1. PCR primers:

| Genes |  | Primer sequence |
| --- | --- | --- |
| *CYP2C9* * 2 | Wildtype primer | GGGCTTCCTCTTGAACGCG |
|  | Mutant primer | GGGCTTCCTCTTGAACGCA |
|  | Universal primer | TGAAACCCATAGTGGTGCTGC |
| *CYP2C9* * 3 | Wildtype primer | CAGGCTGGTGGGGAGAAGGTCGAT |
|  | Mutant primer | CAGGCTGGTGGGGAGAAGGTCGAG |
|  | Universal primer | CTTGCCAAGCTGACCACTTTATCACCAA |
| *VKORC1*-1639*G >A* | Wildtype primer | GACCTGAAAAACAACCATTGGACA |
|  | Mutant primer | GACCTGAAAAACAACCATTGGACG |
|  | Universal primer | CCTTGCTGCCCACGCCATAAAC |

1. PCR amplification system:

For each sample, amplification reaction is performed in wildtype and mutant tubes, respectively. Wildtype and universal primers are added into wildtype tubes, mutant and universal primers are added into mutant tubes.

20μL reaction system includes PCR buffer solution 2μL, 10 mmol·L^-1^ dNTP 0. 4μL, 10 mmol·L^-1^ ARMS primer each 0. 25μL, 50 ng genomic DNA 0. 5μL and Taq DNA polymerase 1.5 U. Finally, double-distilled deionized water is added up to 20μL.

1. PCR amplification conditions:

PCR reaction conditions: 94℃ pre-denaturing 5 min, 94℃ denaturing 30s, 60℃ annealing 30s and 72℃ extension 45s. After 34 cycles, 72℃ re-extension 7 min and lowering temperature to 4℃ for termination. After running 2% agrose gel electrophoresis, the results are interpreted.

At the end of trial, 10% random samples are sent to an accredited central laboratory for testing. And quality control is enforced for genotypic typing during the trial.

# Supplementary Figures


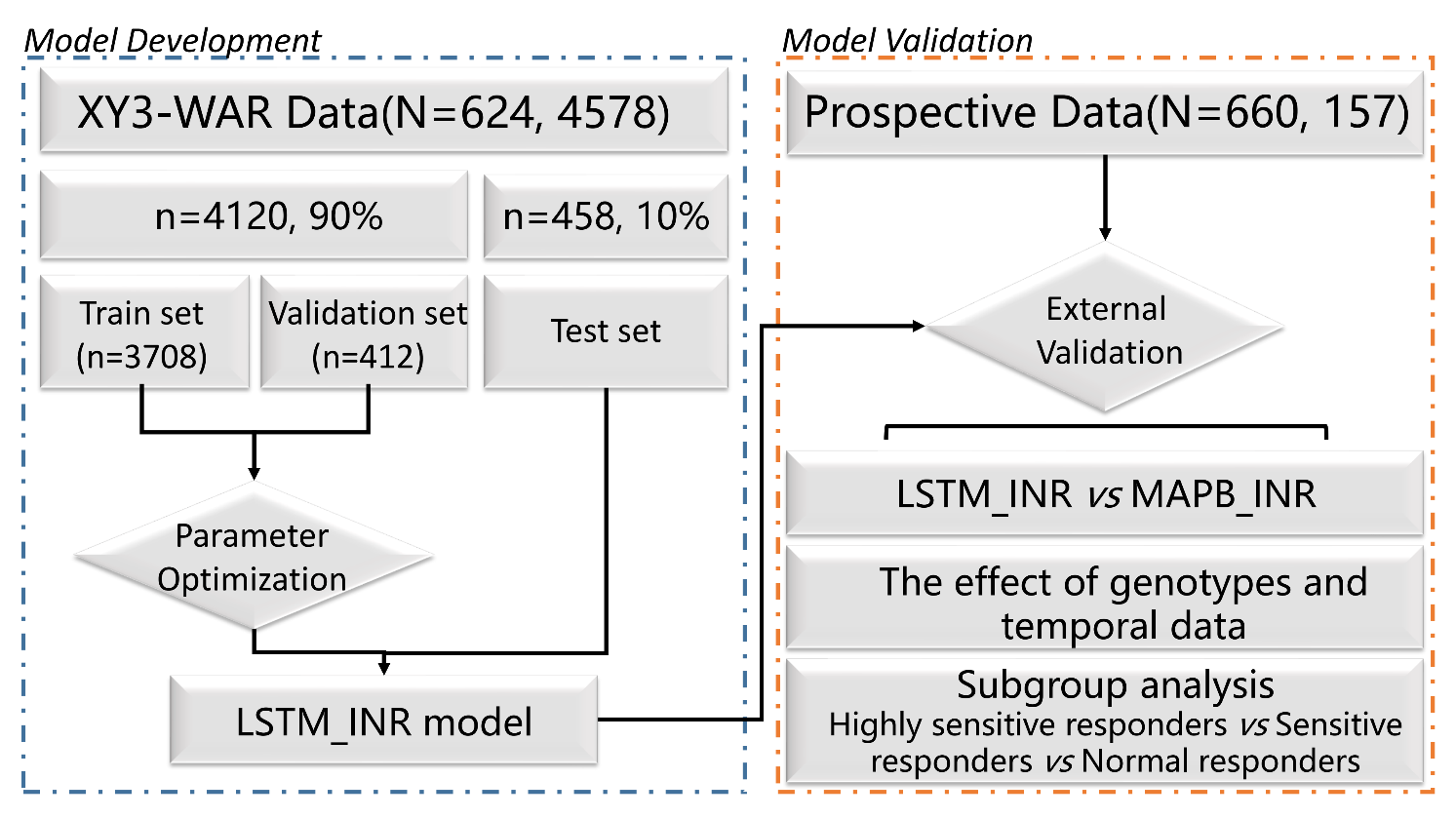


**Supplementary Figure 1.** Flow chart of the analysis used for model development and model validation.

**
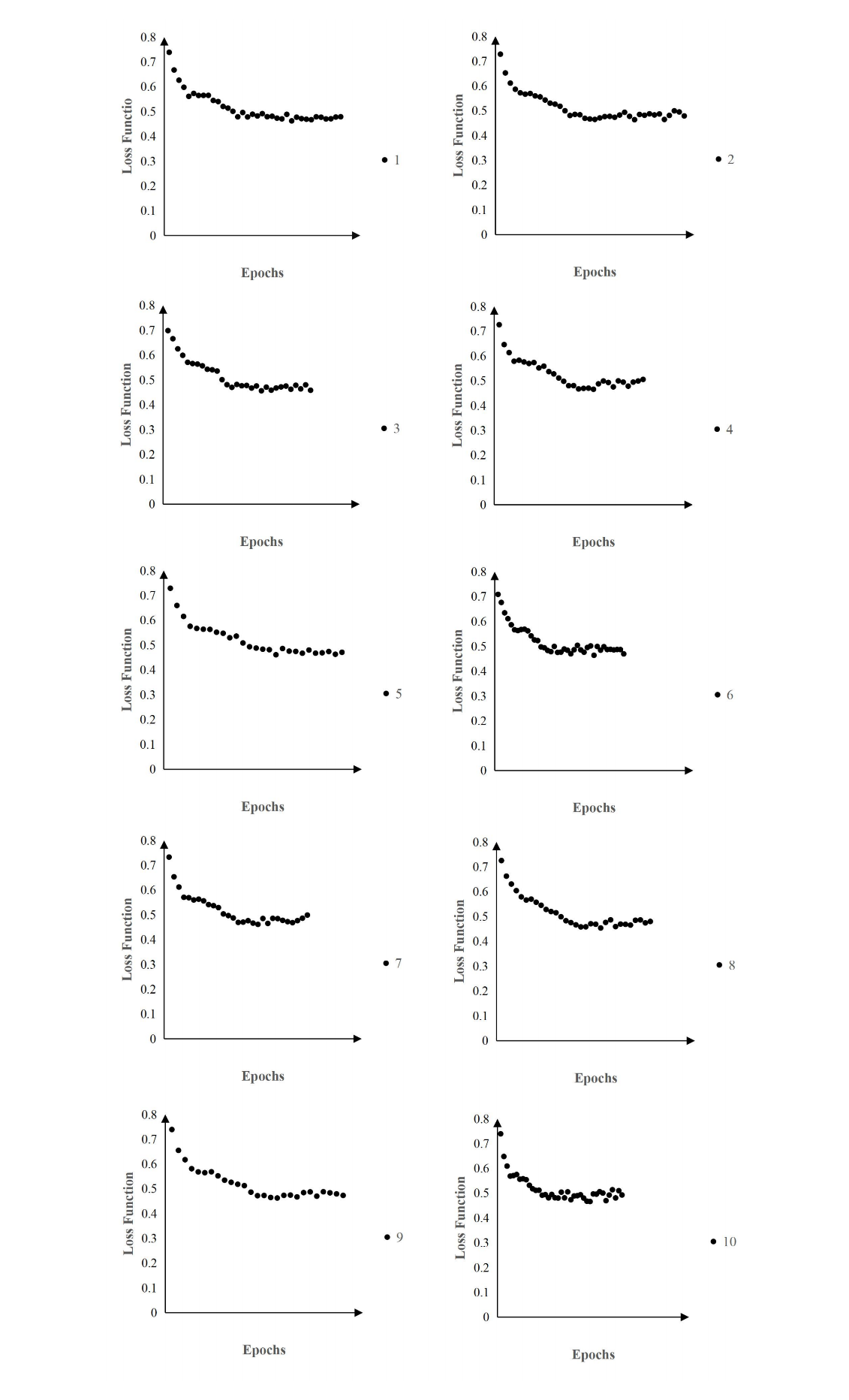
**

**Supplementary Figure 2.** Trend graphs of loss function for 10-fold cross validation. Loss function means mean square error of each epoch.


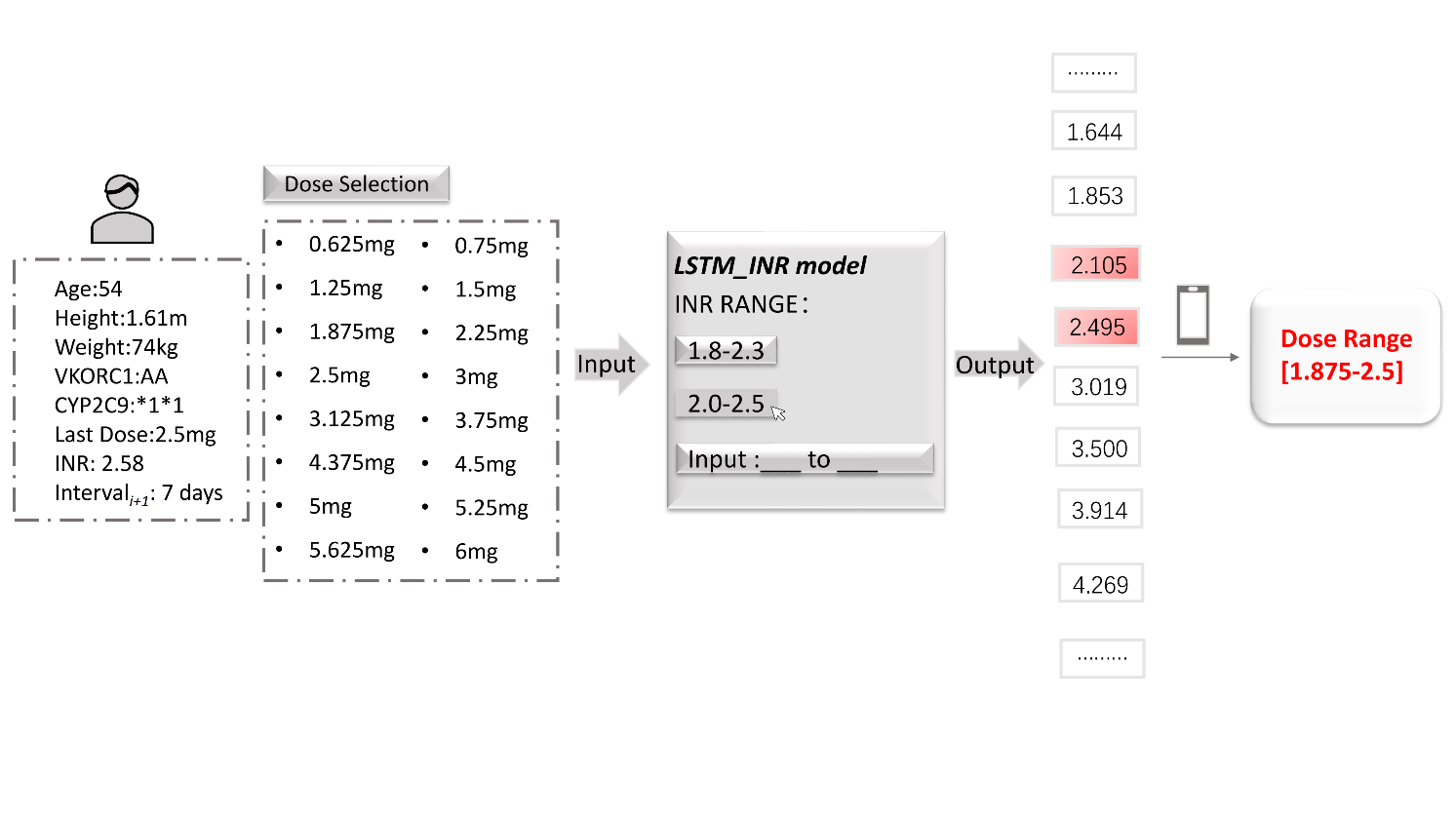


**Supplementary Figure 3.** Flow chart of dose simulation based on LSTM_INR.


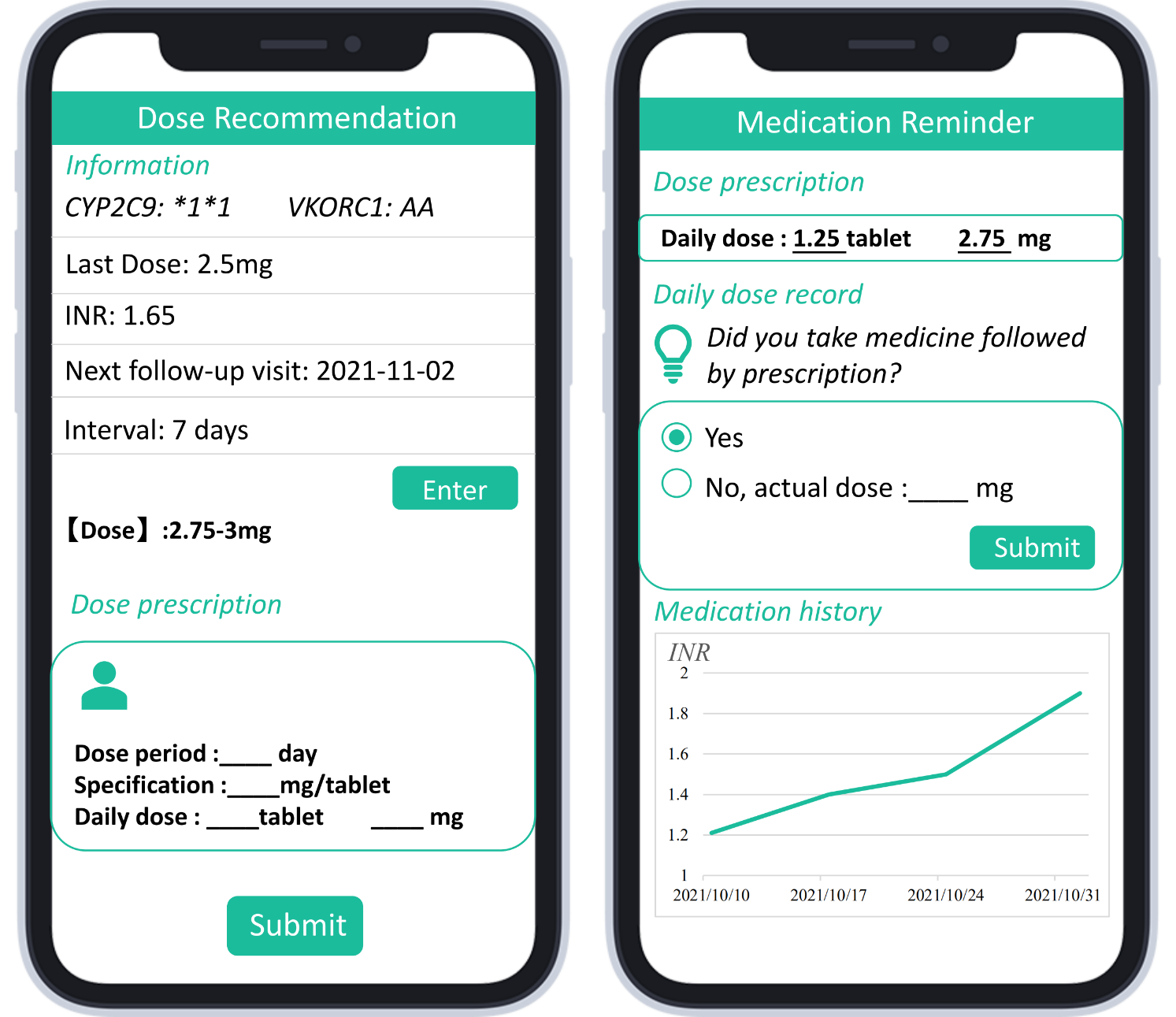


**Supplementary Figure 4.** Function diagram of AI-WAR.
